# Altered dynamic spontaneous neural activity in minimal hepatic encephalopathy

**DOI:** 10.3389/fneur.2022.963551

**Published:** 2022-08-19

**Authors:** Jie-Ru Guo, Jia-Yan Shi, Qiu-Yi Dong, Yun-Bin Cao, Dan Li, Hua-Jun Chen

**Affiliations:** ^1^Department of Gastroenterology, Fujian Medical University Union Hospital, Fuzhou, China; ^2^Department of Radiology, Fujian Medical University Union Hospital, Fuzhou, China

**Keywords:** amplitude of low-frequency fluctuation, dynamic, spontaneous neural activity, minimal hepatic encephalopathy, cognition

## Abstract

**Background and aims:**

Abnormal regional neural activity has been identified by the analysis of the static amplitude of low-frequency fluctuation (ALFF) in the setting of minimal hepatic encephalopathy (MHE). Brain activity is highly dynamic. This work sought to evaluate the temporal variability of ALFF to reveal MHE-related alterations in the dynamics of spontaneous neural activity.

**Methods:**

A total of 29 healthy controls and 49 patients with cirrhosis [including 20 patients with MHE and 29 patients without MHE (NHE)] who underwent resting-state functional magnetic resonance imaging and Psychometric Hepatic Encephalopathy Score (PHES) examination were enrolled in this investigation. Utilizing a sliding-window approach, we calculated the dynamic ALFF (dALFF) variability to reflect the temporal dynamics of regional neural activity. An analysis of the correlation between dALFF variability and PHES was performed, and receiver operating characteristic (ROC) curve analysis to determine the potential of the dALFF variability index in identifying MHE was completed.

**Results:**

The dALFF variability in the bilateral precuneus/posterior cingulate gyrus and left middle frontal gyrus progressively decreased from NHE to MHE group. In cirrhotic patients, the value of dALFF variability in the bilateral precuneus/posterior cingulate gyrus was positively correlated with their neurocognitive performance (*r* = 0.383 and *P* = 0.007). The index of dALFF variability in the bilateral precuneus/posterior cingulate gyrus could be used to distinguish NHE and MHE patients, with moderate power (area under the ROC curve = 0.712 and *P* = 0.012).

**Conclusion:**

Our findings highlight the existence of aberrant dynamic brain function in MHE, which could underlie the neural basis of cognitive impairments and could be associated with the development of the disease. Analyzing dALFF could facilitate new biomarker identification for MHE.

## Introduction

Minimal HE (MHE), as the initial stage of hepatic encephalopathy (HE), is characterized by cognitive impairments, such as a decline in working memory, visuospatial disability, and attention deficit (1). Patients with MHE are at greater risk for experiencing traffic accidents, falls, and work inefficiency, which can subsequently affect their quality of life (2–4). Furthermore, MHE predicts the risk of developing overt HE (a more severe HE phase in which neurological symptoms manifest), and it is linked to a poor prognosis (5). Thus, early detection and appropriate management of MHE are of great clinical significance because they help to prevent further deterioration and hospital admissions, potentially reducing the socioeconomic burden associated with MHE (6, 7).

Resting-state functional magnetic resonance imaging (rs-fMRI) is used to measure low-frequency fluctuations in the blood oxygen level–dependent (BOLD) signal, and it can be utilized to examine brain function (8). To our knowledge, with the advantages of it being convenient, non-invasive, easily accepted by subjects, and having high temporal and spatial resolution, rs-fMRI has been broadly utilized for exploring the mechanisms of neurocognitive impairment (9–11). In fact, rs-fMRI has already helped to further our knowledge of the neuropathological mechanisms underlying MHE (12). In their study, Chen et al. (13) utilized regional homogeneity (ReHo), an index reflecting the synchronization of local neural activity, to examine abnormalities in the brain activity in MHE patients, and they found that reduced ReHo in the cuneus and adjacent precuneus correlated with a worse degree of neurological performance. Ni et al. (14) noted decreases in ReHo in the supplementary motor area and the cuneus in parallel with the development of MHE. In addition, the amplitude of low-frequency fluctuation (ALFF) has also been broadly utilized as an index for the intensity of spontaneous brain activity to examine regional neural activity in MHE (15). Zhong et al. (16) suggested previously in their study that a lowered ALFF index in the precuneus and the medial prefrontal cortex could be a potential biomarker for MHE diagnosis, while another rs-fMRI study by Qi et al. (17) demonstrated that patients with MHE showed reduced ALFF in the regions primarily involving the default-mode network (DMN) and increased ALFF in the cerebellum and middle temporal gyrus, respectively. Chen et al. (18) also determined that MHE patients exhibit decreased ALFF in the DMN, including the posterior cingulate cortex, precuneus, and medial prefrontal cortex. Altered ALFF index have been previously proven by some investigators to be associated with neurocognitive performance in patients with MHE (16–18).

Importantly, however, most of these studies on rs-fMRI in MHE adopted the assumption that brain activity is stagnant throughout scanning (19). Instead, it has been indicated that the brain dynamically reacts and adjusts to external or internal stimuli over multiple time-scales (20). Thus, the traditional static ALFF analysis cannot acquire the entire range of spontaneous brain activity (11, 21). Enter the dynamic ALFF (dALFF) analysis, which, as an extension of ALFF, subdivides the whole time series of BOLD signals into multiple slices, and then it quantifies the ALFF in every slice (22). Thus, dALFF analysis could reflect the time-varying patterns of brain activity (23). Because of its simplicity and ease of implementation (20), this analysis has been broadly applied to detect aberrant dynamics of brain activity and reveal pertinent pathophysiological mechanisms in several brain disorders, including Alzheimer's disease (11), Parkinson's disease (24), generalized anxiety disorder (22), and major depressive disorder (19).

It has recently been suggested that there are abnormalities in the dynamic characteristics of brain activities in MHE patients (25). For example, Chen et al. (26) documented aberrant dynamic functional connectivity (FC) within the DMN in MHE patients. In addition, Cheng et al. (21) found that the reduction of the dynamic FC value in the right insula correlated with neurocognitive decline in the MHE. Zhang et al. (25) demonstrated that abnormal dynamic FC within the DMN and the salience network were associated with attention deficit and visual memory dysfunction in MHE patients, suggesting that dynamic FC analysis could provide powerful features for the individual diagnosis of MHE. Although the above studies suggest that dynamic FC alterations effectively reflect the mechanisms underlying MHE (26), these studies focused on the dynamic FC between different brain regions and did not explore MHE-related change in the dynamics of regional neural activity. In addition, it has been verified that regional neural activity itself exists with substantial fluctuations (27). Thus, this study sought to utilize dALFF analysis to examine the dynamic characteristics of regional neural activity in MHE patients and to explore the relationship between changes in dALFF and patients' cognitive impairment.

## Methods

### Participants

This study was approved by the Research Ethics Committee of the Fujian Medical University Union Hospital, China, and each participant provided written informed consent before enrollment. A total of 29 healthy controls (HCs) and 49 patients with cirrhosis [including 20 patients with MHE and 29 patients without MHE (NHE)] were included in this investigation. Patients were ineligible for inclusion if they (1) were diagnosed with current overt HE or another neuropsychiatric condition, (2) received psychotropic medications or had a history of drug abuse, (3) were diagnosed with an uncontrolled endocrine or metabolic disorder (e.g., thyroid dysfunction), or (4) had abused alcohol in the 6 months leading up to the study. Study participants were matched across the 3 groups (HC vs. NHE vs. MHE) in terms of age (50.8 ± 9.3 vs. 52.4 ± 9.9 vs. 50.4 ± 8.8 years; *P* = 0.726), gender (9 women and 20 men vs. 4 women and 25 men vs. 3 women and 17 men; *P* = 0.208), and education level (9.3 ± 3.0 vs. 8.2 ± 3.1 vs. 8.9 ± 2.8 years; *P* = 0.393). In the NHE group, 18 patients had Child–Pugh stage A and 11 patients had Child–Pugh stage B; while 3, 12, and 5 patients in the MHE group had Child–Pugh stages A, B, and C, respectively. In the NHE group, the etiology of liver cirrhosis included hepatitis B virus (21 cases), hepatitis B virus and alcoholism (2 cases), alcoholism (4 cases), and other causes (2 cases), while, in the MHE group, the etiology of cirrhosis included hepatitis B virus (12 cases), hepatitis B virus and alcoholism (2 cases), alcoholism (4 cases), and other causes (2 cases).

### Neurocognitive assessment

A Psychometric Hepatic Encephalopathy Score (PHES) examination was conducted to complete the neurocognitive assessment. The PHES consists of digit symbol test, number connection tests (A and B test), serial dotting test, and line-tracing test, and information on the use of the PHES tests in diagnosing MHE have previously been described (28).

### MRI data acquisition

Rs-fMRI data were gathered utilizing a 3.0-Tesla MRI scanner (Siemens, Verio, Germany), with the echo planar imaging sequence and the following parameter settings: repetition time, 2.0 s; echo time, 0.025 s; field of view, 240 mm^2^; matrix, 64 × 64; flip angle, 90°; slice thickness, 4 mm; number of axial slices, 35; and number of volume, 180. Patients were told to close their eyes, avoid thinking about a specific issue, and to refrain from moving their head. To acquire T1-weighted structural images, a magnetization-prepared rapid gradient echo (MPRAGE) sequence was used, and the following parameters were applied: repetition time, 1.9 ms; echo time, 2.48 ms; field of view, 256 mm^2^; matrix, 256 × 256; flip angle, 9°; slice thickness, 1.0 mm; and number of sagittal slices, 176.

### Functional MRI data preprocessing

Rs-fMRI data were preprocessed using Statistical Parametric Mapping software (http://www.fil.ion.ucl.ac.uk/spm) and the Data Processing and Analysis of Brain Imaging toolbox (DPABI, http://rfmri.org/DPABI). The processing steps included the following: (1) discard of the first 10 functional volumes; (2) slice timing; (3) realignment; (4) spatial normalization of functional images; (5) smoothing with a Gaussian kernel (full-width at half maximum = 4 mm); (6) regressing out nuisance covariates (including the estimated motion parameters based on the Friston-24 model, white matter signal, and cerebrospinal fluid signal); and (7) linear detrending and band-pass temporal filtering (0.01–0.08 Hz). During data preprocessing, the parameters of head motion were estimated by firstly calculating the translation in the *x, y*, and *z* directions and the angular rotation on every axis for every functional volume, then calculating the frame-wise displacement (FD), which an index reflecting the volume-to-volume head position movements. In the current study, no participant was determined to have head motion of > 2 mm translation or > 2 degrees of rotation. During the normalization step, the individual T1-weighted images, which were co-registered with the mean image of functional data in advance, were segmented and normalized according to the Montreal Neurological Institute (MNI) space by applying a high-level non-linear warping algorithm [i.e., the Diffeomorphic Anatomical Registration Through Exponentiated Lie algebra (DARTEL) approach] (29). Then, functional images were spatially normalized to MNI space and resampled into a 3-mm cubic voxel using the deformation parameters derived from the above procedure.

### Dynamic ALFF analysis

The dynamic ALFF analysis was conducted with the Temporal Dynamic Analysis (TDA) toolkit, implemented in the DPABI software, utilizing a sliding-window approach with the following parameters: window size, 32 TR (= 64 s); sliding step, 1 TR (= 2 s); window number, 139; and window type, Hamming (30, 31). Within each window, the ALFF map was computed. In short, utilizing the fast Fourier transform technique, the time courses were transformed from the time domain to a frequency domain. Then, from the frequency domain, the power spectrum was achieved. The square root of the power spectrum was computed and averaged across a range of 0.01–0.08 Hz at each voxel. The acquired square root value was then taken as the ALFF. Thus, a series of ALFF maps were acquired for every subject. To determine the temporal variability of ALFF (i.e., the dALFF variability), the standard deviation (SD) values of all of the ALFF maps across windows were computed. After that, the dALFF variability of every voxel was transformed into a *Z*-score by subtracting the mean and dividing by the SD of the global value (22).

### Statistical analysis

The one-way analysis of variance (ANOVA) was performed for the comparisons of demographic variables (i.e., age and education level), while the chi-squared test was conducted to compare categorical variables (i.e., gender), with the statistical threshold of *P* < 0.05.

To examine the distribution pattern of dALFF variability in every group, the value of dALFF variability was averaged at every voxel across subjects within HC, NHE, and MHE groups, respectively. Using the “Statistical Analysis Module” in the DPABI software, a voxel-wise ANOVA was conducted to detect differences in dALFF variability between the 3 groups, with the statistical threshold of *P* < 0.05, which was corrected by the Gaussian Random Field (GRF) theory (with the voxel-level *P* < 0.001 and the cluster-level *P* < 0.05). Several covariates, such as age, gender, education level, and FD value, were included in the ANOVA.

After the ANOVA, the regions that showed significant dALFF variability differences were identified; then, we calculated the average dALFF variability values in these regions for each subject and compared them between every two groups by the independent-sample *t* test. Using Spearman's correlation analysis, the relationship between the dALFF variability index and PHES result was examined among cirrhotic patients. Using the above-mentioned mean dALFF variability value as the index, a receiver operating characteristic (ROC) curve analysis was complete to evaluate the success of discrimination between the NHE and MHE groups. Finally, the area under the ROC curve (AUC) was estimated. The above procedures were conducted using the SPSS 22.0 software (SPSS, Inc., Chicago, IL, USA).

### Validation analysis

As previous studies have suggested, brain structural differences may confound the results of ALFF analysis (32). To explore this possible effect, the dALFF analysis by taking voxel-wise gray matter volume (GMV) as covariates was additionally performed, using the “Statistical Analysis Module” in the DPABI software. Based on structural T1-weighted images, GMV maps were obtained using the DARTEL method (29).

In consideration of existing controversy regarding global signal regression (GSR) in rs-fMRI data preprocessing (33, 34), an analysis with the addition of GSR was conducted as well in order to further verify our results. Also, in consideration of the controversy about parameter-setting in the sliding-window approach (35), the following steps were additionally completed to investigate the influence of the choice of distinct window length, sliding step, and window type: (1) the analyses were performed with different window lengths (24 TR, 32 TR, 40 TR, and 50 TR); (2) the analyses were performed with different sliding steps (1 TR, 2 TR, and 4 TR); and (3) the analyses were performed using different window types (Hamming and rectangular windows).

A static ALFF (sALFF) analysis was also conducted in order to determine whether sALFF and dALFF analyses yielded similar or complementary information about the neuropathological mechanisms underlying MHE. For each participant, an sALFF map was created using the DPABI toolbox, and the individual sALFF maps were standardized by dividing the global mean ALFF value (32).

## Results

### Neurocognitive assessment result

Neurocognitive assessment results are summarized in Table 1. Compared to the HC and NHE groups, patients with MHE showed lower PHES scores, indicating worse neurocognitive performance. To be specific, MHE patients required more time to finish the number connection tests and serial dotting test; acquired lower scores in the digit symbol test; and did worse on the line-tracing test. Further, the neurocognitive dysfunction was worse in the MHE group relative to the NHE group.

**Table 1:** Results of neurocognitive assessment.

	**HCs**	**NHE patients**	**MHE patients**	***P*-value**
Digit symbol test (raw score)	46.9 ± 10.7	41.5 ± 12.5	27.2 ± 8.8^*, #^	<0.001
Number connection test A (seconds)	36.5 ± 11.7	38.7 ± 10.7	56.3 ± 17.7^*, #^	<0.001
Number connection test B (seconds)	60.1 ± 26.8	73.6 ± 27.3	128.8 ± 58.3^*, #^	<0.001
Serial dotting test (seconds)	40.9 ± 7.2	46.8 ± 9.3^†^	61.9 ± 12.7^*, #^	<0.001
Line tracing test (raw score)	113.7 ± 19.0	145.6 ± 35.3^†^	184.5 ± 34.5^*, #^	<0.001
Psychometric Hepatic Encephalopathy Score	0.83 ± 2.05	−0.66 ± 2.22^†^	−8.10 ± 2.95^*, #^	<0.001

HC, healthy control; MHE, minimal hepatic encephalopathy; NHE, cirrhotic patients without MHE.

The ^*^ denotes the significant difference between the HC and MHE groups, the ^#^ denotes the significant difference between the NHE and MHE groups, and the ^†^ denotes the significant difference between the HC and NHE groups.

### Result of DALFF analysis

**Figure 1:**
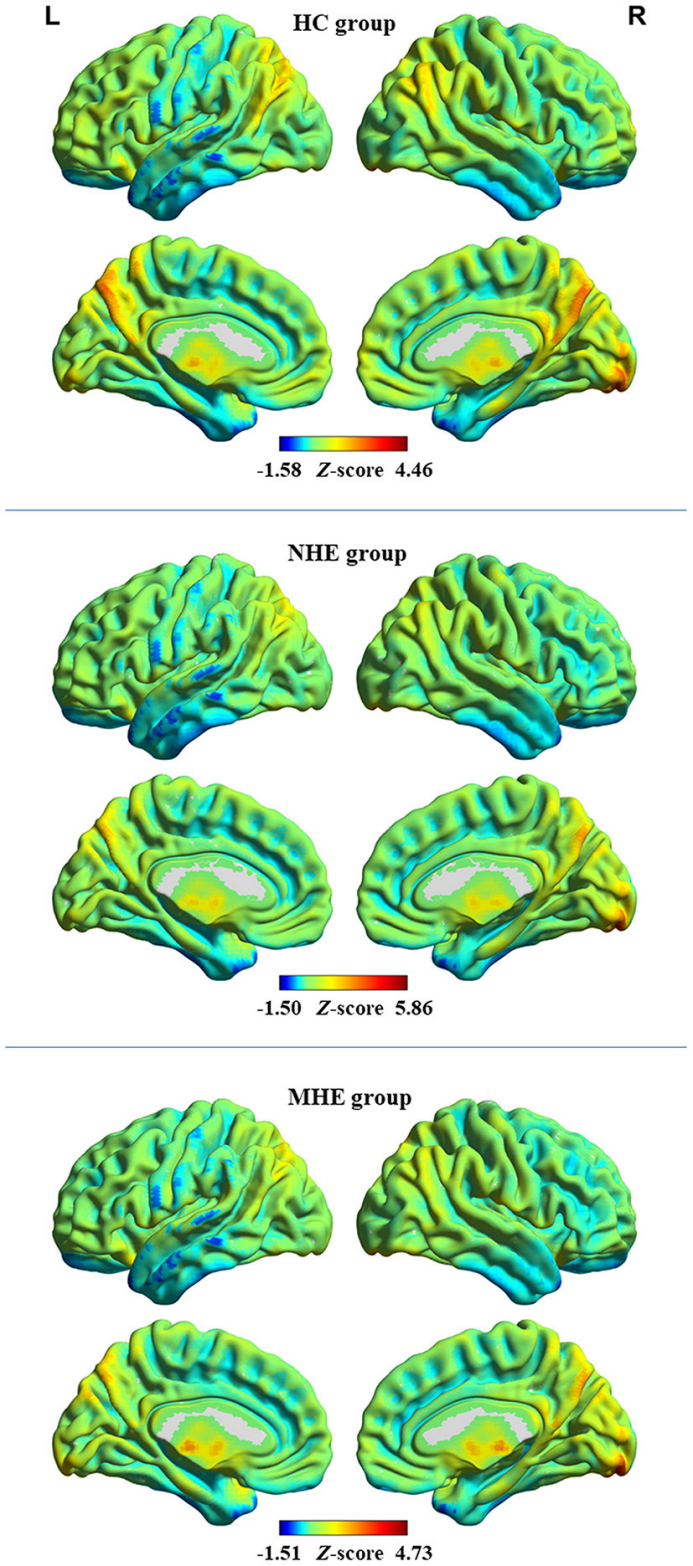
The distribution pattern of dALFF variability in the healthy control (HC) group, group of patients with minimal hepatic encephalopathy (MHE), and group of patients without MHE (NHE). The red and blue colors, respectively, indicate high and low dALFF variability. “L” denotes the left hemisphere and “R” denotes the right hemisphere.

The distribution pattern of dALFF variability in each group is shown in Figure 1. In the HC group, relatively greater dALFF variability was observed in several regions, including the precuneus, posterior cingulate cortex, parietal–temporal junction, prefrontal cortex, and insular cortex, and relatively less dALFF variability was observed in the sensorimotor cortex, temporal lobe, and subcortical regions. Further, cirrhotic patients in the NHE and MHE groups showed similar spatial distributions of dALFF variability.

**Figure 2:**
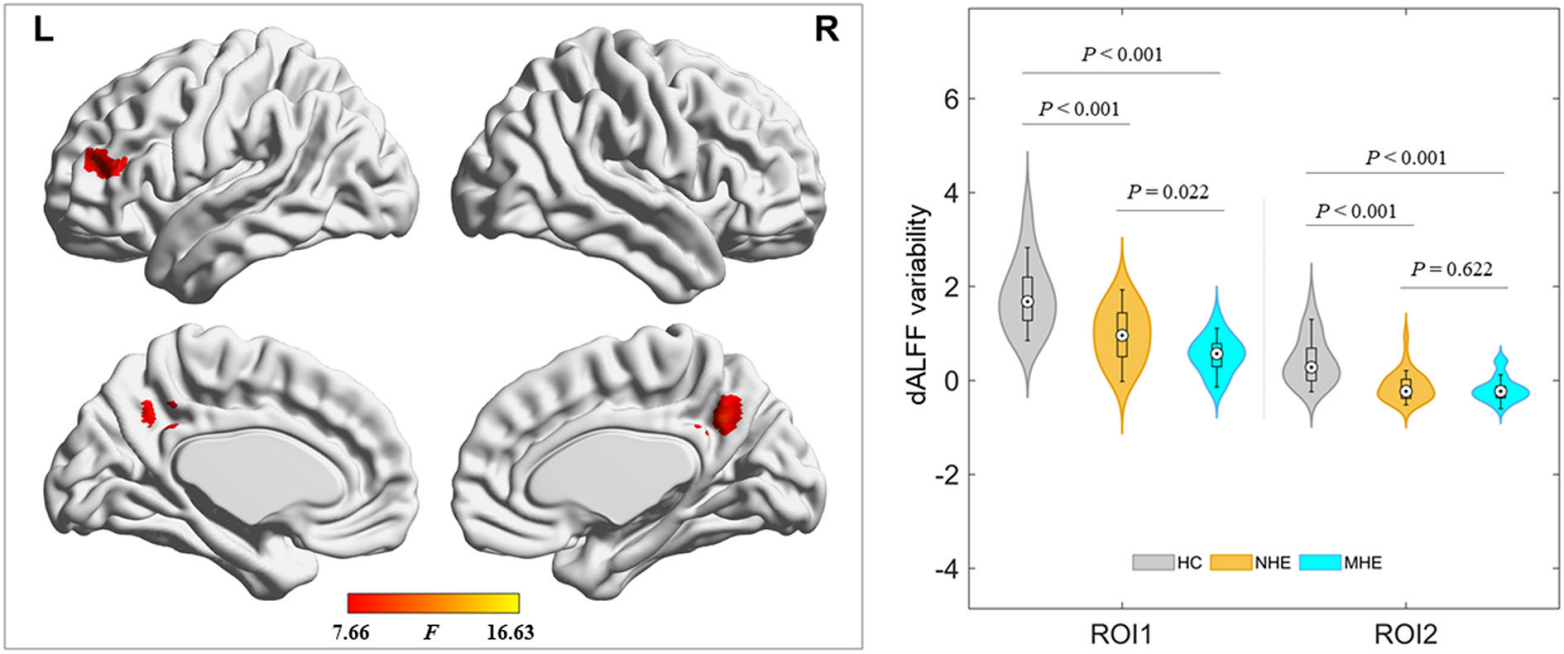
Brain regions where significant differences in dALFF variability were located across the 3 groups. The combination of violin and box plots shows the distribution and between-group differences of mean dALFF values in these regions. ROI1, bilateral precuneus and posterior cingulate gyrus; ROI2, left middle frontal gyrus. HC, healthy control; MHE, minimal hepatic encephalopathy; NHE, patients without MHE. dALFF, dynamic amplitude of low-frequency fluctuation.

The ANOVA results are shown in Figure 2 and Table 2. Among the 3 study groups, the areas with significant dALFF variability differences involved 2 regions. Of these, region of interest (ROI) 1 was located in the bilateral precuneus and posterior cingulate gyrus, and ROI2 was located in the left middle frontal gyrus. The mean dALFF variability in both regions was decreased in the MHE group compared to the HC group. Compared to the NHE group, MHE patients showed significantly decreased dALFF variability in ROI1.

**Table 2:** Brain regions with significant differences in dALFF variability.

**Regions**	**Voxels**	**Brodmann area**	**MNI coordinates**			**Peak *F* value**	**Mean dALFF value within regions**		
			**x**	**y**	**z**		**HC group**	**NHE group**	**MHE group**
Bilateral precuneus and posterior cingulate gyrus	20	7/31	0	−51	36	16.6	1.80 ± 0.76	0.96 ± 0.57	0.54 ± 0.39
Left middle frontal gyrus	11	10	−39	42	18	13.5	0.42 ± 0.52	−0.13 ± 0.31	−0.19 ± 0.26

dALFF, dynamic amplitude of low-frequency fluctuation; MNI, Montreal Neurological Institute; HC, healthy control; MHE, minimal hepatic encephalopathy; NHE, cirrhotic patients without MHE.

### Result of correlation analysis

**Figure 3:**
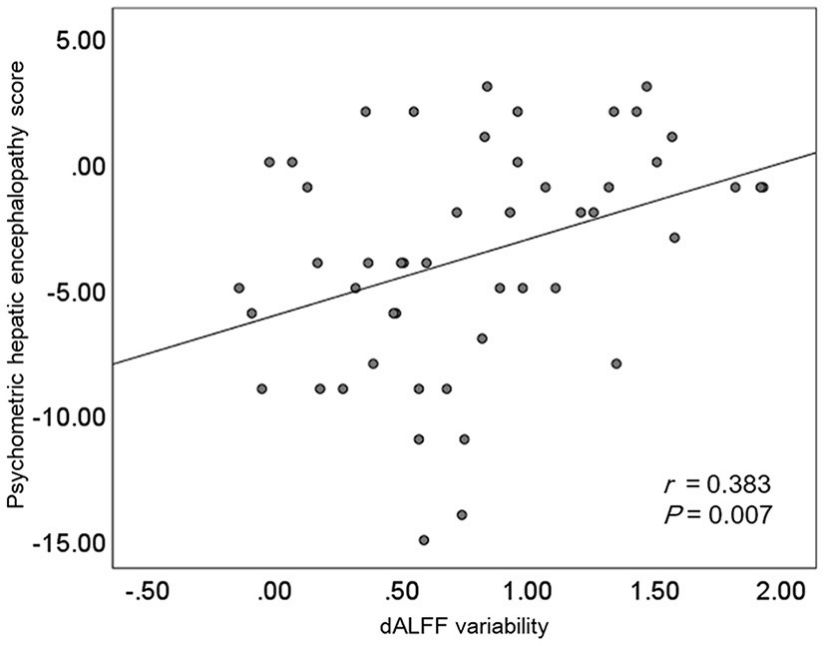
The results of correlation analyses. dALFF, dynamic amplitude of low-frequency fluctuation.

Results of the correlation analyses are shown in Figure 3. A significantly positive correlation between the PHES result and the mean dALFF variability in the bilateral precuneus and posterior cingulate gyrus was observed among cirrhotic patients (*r* = 0.383 and *P* = 0.007), whereas no significant correlation between the mean dALFF variability in the left middle frontal gyrus and the PHES result was found.

### Result of ROC curve analysis

**Figure 4:**
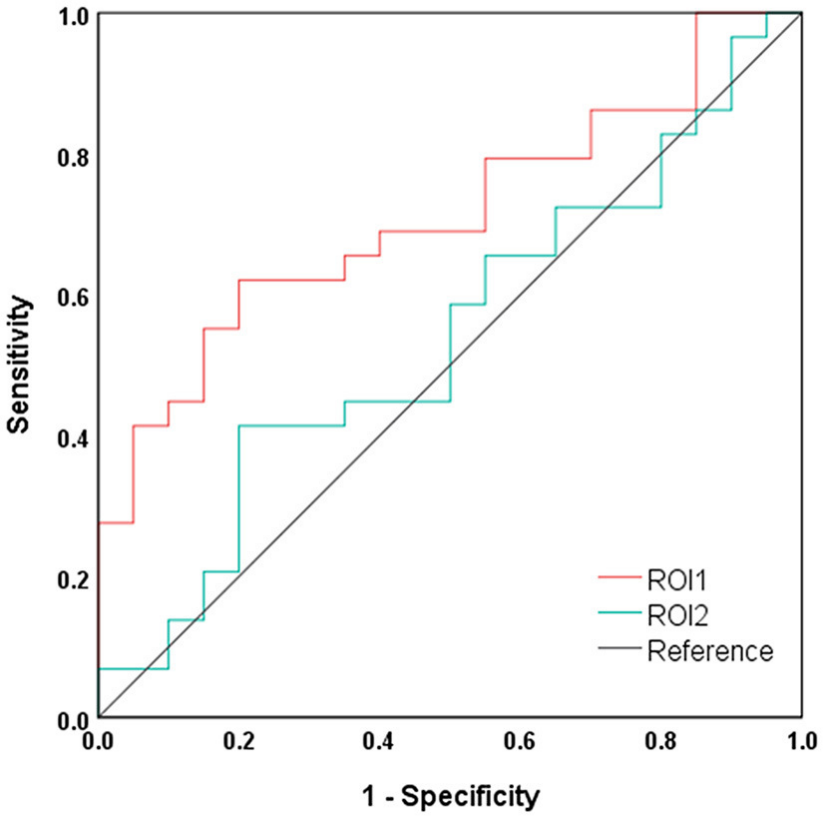
Results of ROC curve analysis. ROI1, bilateral precuneus and posterior cingulate gyrus; ROI2, left middle frontal gyrus.

The results of ROC curve analyses are shown in Figure 4. The index of mean dALFF variability in ROI1 (i.e., the bilateral precuneus and posterior cingulate gyrus) showed moderate power in distinguishing NHE and MHE patients (AUC = 0.712 and *P* = 0.012), whereas that in ROI2 (i.e., left middle frontal gyrus) did not exhibit good discrimination potential (AUC = 0.544 and *P* = 0.604).

### Result of validation analysis

**Figure 5:**
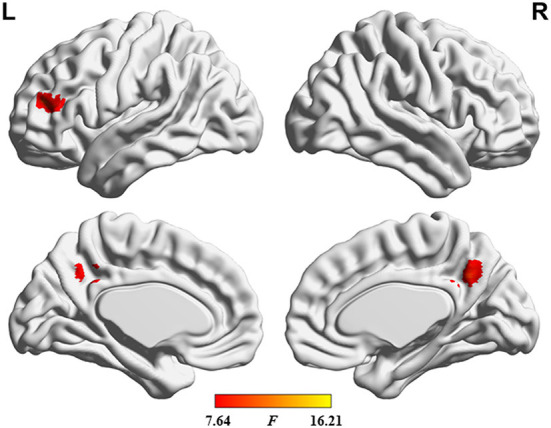
Results of dALFF analysis with GMV as a covariate.

**Figure 6:**
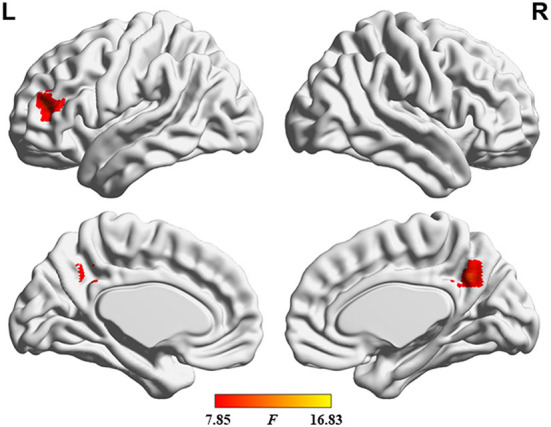
Results of dALFF analysis with the GSR.

**Figure 7:**
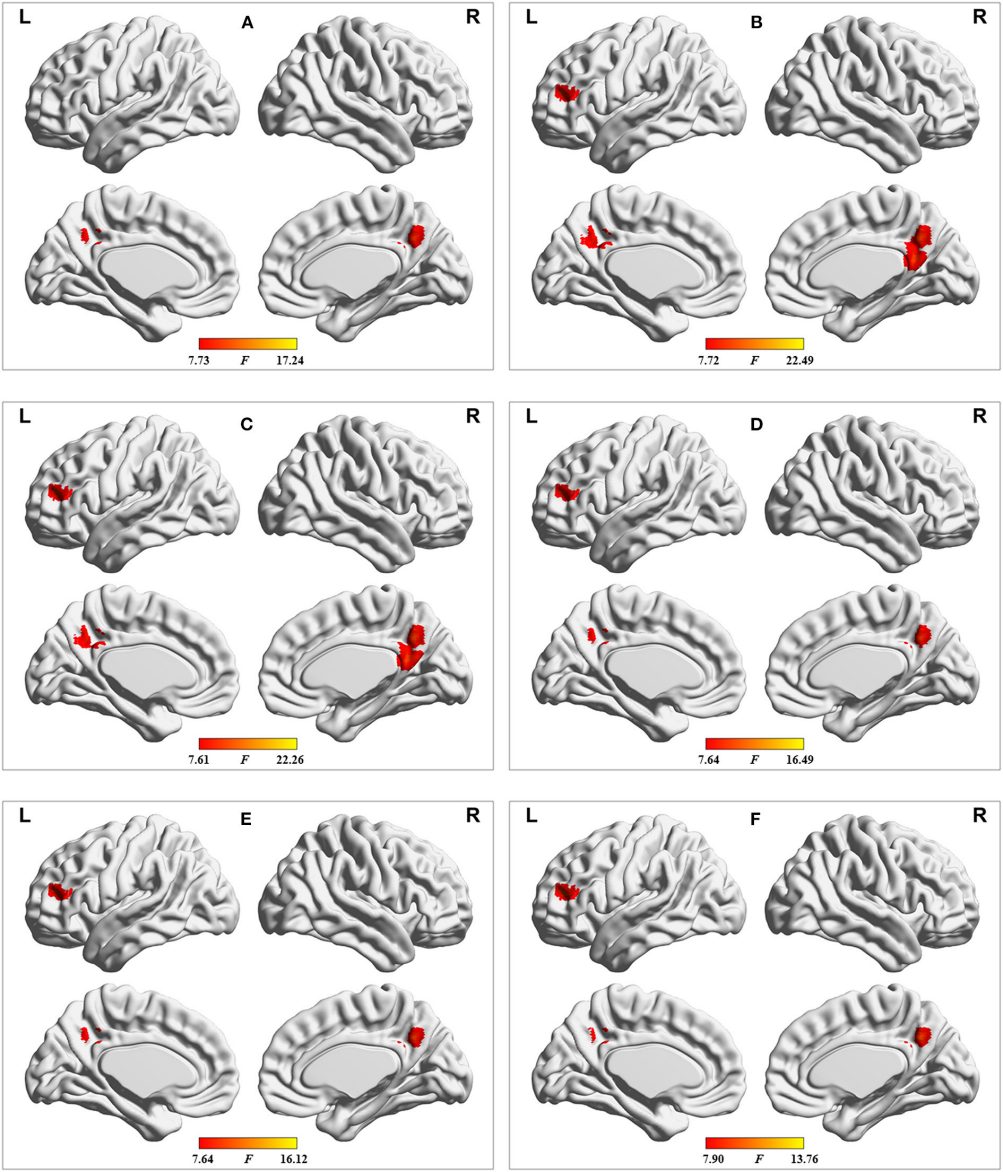
Results of dALFF analyses with the following distinct sliding-window parameter settings: **(A)** window size of 24 TR, sliding step of 1 TR, and Hamming window type; **(B)** window size of 32 TR, sliding step of 2 TR, and Hamming window type; **(C)** window size of 32 TR, sliding step of 4 TR, and Hamming window type; **(D)** window size of 40 TR, sliding step of 1 TR, and Hamming window type; **(E)** window size of 50 TR, sliding step of 1 TR, and Hamming window type; and **(F)** window size of 32 TR, sliding step of 1 TR, and rectangular window type. TR = 2,000 ms.

**Figure 8:**
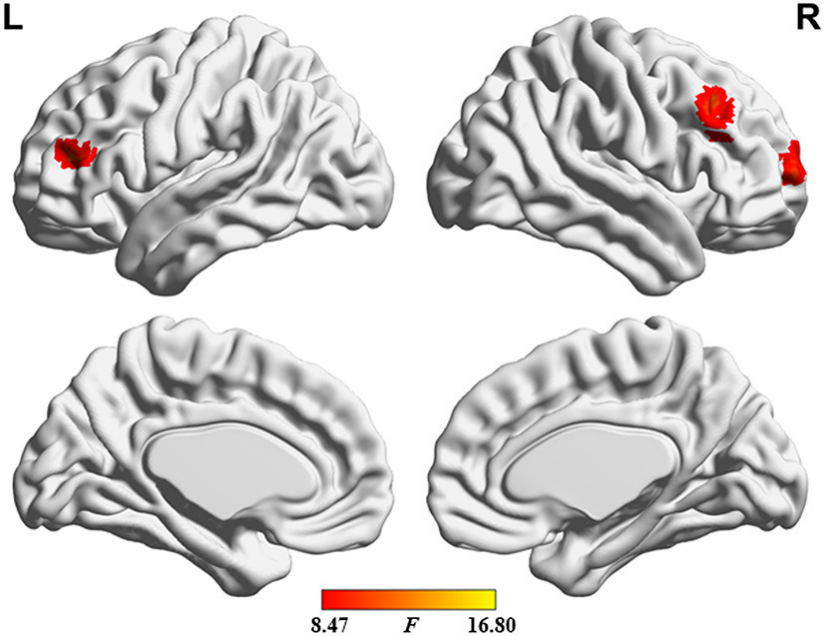
Brain regions where the significant differences in sALFF were found across the 3 groups.

The results of validation analyses are shown in Figures 5–7. Of note, when using GMV as a covariate, the ANOVA analysis showed a similar result as that obtained without GMV as a covariate. Additionally, the major findings were similar when the dALFF analyses without and with GSR were performed. Finally, the distinct sliding-window parameter settings did not result in a significant change in the results of ANOVA. These findings of the dALFF analyses suggest the reliability and reproducibility of our results. In addition, the sALFF analysis revealed that the group difference involved the left middle frontal gyrus and right superior and middle frontal gyrus (Figure 8). The distribution of group difference was similar to that noted in the dALFF analysis, to a certain extent; meanwhile, it was documented that dALFF analysis produced more key information (e.g., altered dALFF in the bilateral precuneus and posterior cingulate gyrus), which may support greater insight into the neuropathological mechanisms underlying MHE.

## Discussion

To our knowledge, dALFF variability was utilized to reflect the abnormality of dynamic brain activity in MHE in this study for the first time. Our analysis showed that the distribution pattern of dALFF variability in HC group was consistent with prior report (22). Our findings included progressively decreased dALFF variability in the bilateral precuneus and posterior cingulate gyrus and the left middle frontal gyrus in the MHE group relative to the NHE group, indicating that the dALFF analysis could help provide an index for monitoring disease development. A significant correlation between patients' neurocognitive performance and dALFF variability in the bilateral precuneus and posterior cingulate gyrus was noted, indicating that dALFF variability may be an alternative index to establish the neurophysiological characteristics of MHE. Moreover, our results showed that the index of dALFF variability in the bilateral precuneus and posterior cingulate gyrus had moderate power in distinguishing NHE and MHE patients, supporting its potential as a diagnostic biomarker for MHE.

The complicated nature of brain activity may be reflected by electroencephalography (EEG) and also informed by the lower temporal resolution of rs-fMRI (36). Concurrent rs-fMRI–EEG studies have demonstrated that the variability of dALFF closely correlates with the EEG power (37). It is important to note that slowing of the EEG rhythm (indicated by an enhanced θ power and reduced α and β powers) and suppression of EEG signal variability have been documented in cirrhotic patients with different degrees of neurocognitive impairment (38). Thus, it is unsurprising that a reduction in dALFF variability was observed in MHE patients in the current study.

Our cirrhotic patients with MHE had decreased dALFF variability in the bilateral precuneus and posterior cingulate gyrus, suggesting the reduced adaptability and efficiency of these regions in responding to the stimuli (37). In accordance with our findings, increasing evidence suggests structural [as indicated by GMV reduction (39)] and functional [as reflected by abnormal FC (40)] abnormalities exist in the precuneus and posterior cingulate gyrus of MHE patients that may be responsible for the patients' cognitive deficits. Further, previous MHE studies incorporating sALFF analysis have consistently noted abnormal neural activities in the precuneus and posterior cingulate gyrus, underlying the neural basis of the cognitive dysfunction viewed in patients (16, 18). The precuneus and posterior cingulate gyrus together comprise the primary node of the DMN, a neurocognition-related network that shows high metabolic activity throughout rest and is thought to be involved in the core process of cognitively demanding tasks like visual processing, attention, and working memory (41–44). As such, prior research suggested the breakdown of intrinsic neural activity within the DMN to be a neuropathological mechanism in MHE (17). Meanwhile, the precuneus participates in the process of visuo-spatial information integration (45), and the posterior cingulate gyrus plays a direct role in regulating the focus of attention (46). It is notable that MHE patients frequently present with reduced visuo-spatial abilities and attentional impairments (47). Hence, it can be inferred that the reduced fluctuation of local neural activity in the precuneus and posterior cingulate gyrus may be associated with the neurobiological basis of these functional deficits in MHE. This speculation could be supported by the observed correlation between the PHES result and the dALFF variability in the bilateral precuneus and posterior cingulate gyrus.

MHE patients also revealed decreased dALFF variability in the left middle frontal gyrus, suggesting a disruption in local brain activity fluctuations (48). It has consistently been documented that resting-state neural activity is abnormal (as reflected by altered ReHo) in the left middle frontal gyrus of MHE patients (49). Moreover, an rs-fMRI study demonstrated the decreased degree centrality (an index quantifying nodal importance in functional integration in the whole-brain network) in the left middle frontal gyrus in cirrhotic patients with early HE, which is correlated with cognitive deficits (28). Research suggests the left middle frontal gyrus is intimately involved in the integration and processing of spatial working memory (50), and the dysfunction of spatial working memory is regarded as an MHE-related characteristic (47). Therefore, lowered dALFF variability in the left middle frontal gyrus may account to some extent for the impairments in spatial working memory noted among patients with cirrhosis and early HE.

Several limitations of this study should be noted. First, the relatively small sample size may limit the power of the statistical analysis, to some extent; second, the heterogeneity of the cirrhotic etiology may produce bias in the classification results because the damage to the brain caused by liver cirrhosis of distinct etiologies can be slightly different (51). Finally, there was a lack of recordings of the cardiac and respiratory fluctuations, which may be aliased into low-frequency fMRI signal fluctuations. The elimination of physiological noises (e.g., the cardiac and respiratory fluctuations) is difficult in rs-fMRI data processing, and the simultaneous recording of these noises could contribute to a more direct correction in a future study.

In conclusion, MHE patients showed reduced temporal variability of dALFF in areas of the brain implicated primarily in visuo-spatial functioning, attention, and spatial working memory. As a result, this study has further validated the fact that abnormal neural activity is an important characteristic of MHE, from a dynamic perspective. The present dALFF analysis shed new insight into the neurobiological basis underlying cognitive dysfunction in patients with MHE and may support imaging biomarker identification to facilitate the diagnosis of MHE.

## Data availability statement

The original contributions presented in the study are included in the article/supplementary material, further inquiries can be directed to the corresponding authors.

## Ethics statement

This study was approved by the Research Ethics Committee of the Fujian Medical University Union Hospital, China. The patients/participants provided their written informed consent to participate in this study.

## Author contributions

J-RG and J-YS: data curation, investigation, formal analysis, and writing-review and editing. Q-YD: data curation, validation, roles, and writing-original draft. Y-BC: data curation, investigation, and formal analysis. DL: supervision, investigation, project administration, roles, and writing-original draft. H-JC: conceptualization, supervision, formal analysis, project administration, writing-review and editing, and funding acquisition. All authors contributed to the article and approved the submitted version.

## Funding

This research was supported by grants from the National Natural Science Foundation of China (No. 82071900), Fujian Provincial Health Technology Project (No. 2021CXA010), and Fujian Province Joint Funds for the Innovation of Science and Technology (No. 2019Y9067).

## Conflict of interest

The authors declare that the research was conducted in the absence of any commercial or financial relationships that could be construed as a potential conflict of interest.

## Publisher's note

All claims expressed in this article are solely those of the authors and do not necessarily represent those of their affiliated organizations, or those of the publisher, the editors and the reviewers. Any product that may be evaluated in this article, or claim that may be made by its manufacturer, is not guaranteed or endorsed by the publisher.

## Abbreviations

HE: hepatic encephalopathy
MHE: minimal hepatic encephalopathy
rs-fMRI: resting-state functional magnetic resonance imaging
BOLD: blood oxygen level–dependent
ReHo: regional homogeneity
ALFF: amplitude of low-frequency fluctuation
DMN: default mode network
dALFF: dynamic amplitude of low-frequency fluctuation
FC: functional connectivity
HC: healthy control
NHE: cirrhotic patients without minimal hepatic encephalopathy
PHES: Psychometric Hepatic Encephalopathy Score
DPABI: Data Processing and Analysis of Brain Imaging Toolbox
FD: frame-wise displacement
MNI: Montreal Neurological Institute
DARTEL: Anatomical Registration Through Exponentiated Lie algebra
SD: standard deviation
ANOVA: analysis of variance
ROC: receiver operating characteristic
AUC: the area under the receiver operating characteristic curve
GMV: gray matter volume
GSR: global signal regression
ROI: region of interest
sALFF: static amplitude of low-frequency fluctuation
EEG: electroencephalography.
